# SK3 in POMC neurons plays a sexually dimorphic role in energy and glucose homeostasis

**DOI:** 10.1186/s13578-022-00907-2

**Published:** 2022-10-09

**Authors:** Meng Yu, Jonathan C. Bean, Hailan Liu, Yang He, Yongjie Yang, Xing Cai, Kaifan Yu, Zhou Pei, Hesong Liu, Longlong Tu, Kristine M. Conde, Mengjie Wang, Yongxiang Li, Na Yin, Nan Zhang, Junying Han, Nikolas A. Scarcelli, Pingwen Xu, Yanlin He, Yong Xu, Chunmei Wang

**Affiliations:** 1 Children’s Nutrition Research Center, Department of Pediatrics, Baylor College of Medicine, One Baylor Plaza, Houston, TX 77030 USA; 2 Division of Endocrinology, Diabetes, and Metabolism, Department of Medicine, The University of Illinois at Chicago, Chicago, IL 60612 USA; 3 Pennington Biomedical Research Center, Brain glycemic and metabolism control department, Louisiana State University, Baton Rouge, LA 70808 USA; 4 Department of Molecular and Cellular Biology, Baylor College of Medicine, One Baylor Plaza, Houston, TX 77030 USA

**Keywords:** POMC neurons, SK current, Sexually dimorphism, Energy and glucose homeostasis

## Abstract

**Background:**

Pro-opiomelanocortin (POMC) neurons play a sexually dimorphic role in body weight and glucose balance. However, the mechanisms for the sex differences in POMC neuron functions are not fully understood.

**Results:**

We detected small conductance calcium-activated potassium (SK) current in POMC neurons. Secondary analysis of published single-cell RNA-Seq data showed that POMC neurons abundantly express SK3, one SK channel subunit. To test whether SK3 in POMC neurons regulates POMC neuron functions on energy and glucose homeostasis, we used a Cre-loxP strategy to delete SK3 specifically from mature POMC neurons. POMC-specific deletion of SK3 did not affect body weight in either male or female mice. Interestingly, male mutant mice showed not only decreased food intake but also decreased physical activity, resulting in unchanged body weight. Further, POMC-specific SK3 deficiency impaired glucose balance specifically in female mice but not in male mice. Finally, no sex differences were detected in the expression of SK3 and SK current in total POMC neurons. However, we found higher SK current but lower SK3 positive neuron population in male POMC neurons co-expressing estrogen receptor α (ERα) compared to that in females.

**Conclusion:**

These results revealed a sexually dimorphic role of SK3 in POMC neurons in both energy and glucose homeostasis independent of body weight control, which was associated with the sex difference of SK current in a subpopulation of POMC + ERα + neurons.

## Introduction

Pro-opiomelanocortin (POMC) neurons in the arcuate nucleus of the hypothalamus (ARH) play essential roles in the regulation of body weight and glucose balance. Ablation of POMC neurons or *Pomc* gene deficiency causes hyperphagia and obesity, while activation of POMC neurons or re-expression of *Pomc* gene inhibits feeding and body weight gain [1–3]. Consistently, mice with ablated POMC neurons develop glucose intolerance [1]. Chronic activation of POMC neurons suppresses hepatic gluconeogenesis, while the inhibition of POMC neurons does the opposite [4]. However, in contrast to POMC neuron functions on energy homeostasis, mice with the deletion of *Pomc* gene from the hypothalamus unexpectedly show improved glucose tolerance in both sexes [5, 6]. This inconsistency of *Pomc* gene functions between body weight and glucose balance suggests that POMC neurons are functionally segregated and regulate glucose balance independent of energy homeostasis.

Sex differences exist in both energy and glucose homeostasis [7–11]. Pre-menopausal women are more protected from obesity-associated metabolic dysregulation compared to aged-matched men [7–10] and female animals have better glucose tolerance than males [12]. Accumulating evidence suggests that POMC neurons are sexually dimorphic, with higher POMC gene expression, higher POMC neuron number and neural activity in female animals [13]. Importantly, POMC neurons play sexually dimorphic roles in both energy and glucose homeostasis [13–15]. Interestingly, only female mice display obesity or diet-induced obesity with POMC neuron-specific disruptions of genes including estrogen receptor α, *Esr1*, (ERα) [14], transcriptionally active p63, *Trp63*, (TAp63) [13], steroid receptor coactivator-1, *Ncoa1*, (SRC-1) [15], Sirtuin 1, *Sirt1*^16^ or signal transducer and activator of transcription 3, *Stat3*, (STAT3) [17]. Coincidently, all these genes promote POMC neuron activity and/or *Pomc* gene expression. Glucose intolerance is developed in male mice with POMC neuron-specific deficiency of leptin receptor, *Lepr*, (LepR) [18], autophagy-related 7, *Atg7*, (ATG7) [19] or Protein Kinase C λ, *Prcki*, (PKCλ) [20] and in female mice with POMC neuron-specific deficiency of liver kinase B1, *Lkb1*, (LKB1) [21] or double deletion of LepR and insulin receptor, *Insr*, (IR) [22], regardless of the body weight change. These results suggest that POMC neurons are functionally heterogeneous and sexually dimorphic in both energy and glucose homeostasis. However, detailed mechanisms for these sub-populations of POMC neurons are not well studied.

The small conductance calcium-activated potassium channel (SK) is a subfamily of Ca^2+^-activated K^+^ channels [23–25]. Activation of SK channels triggers potassium outflux, which contributes to after-hyperpolarization (AHP) [26]. AHP can influence firing frequency and spike frequency adaptation of neurons which finally lead to the inhibition of neuron excitability. Typically, a functional SK channel contains a homomeric or heteromeric tetramer of SK subunits, which are encoded by genes *Kccn1* (SK1), *Kccn2* (SK2), *Kccn3* (SK3) and *Kccn4* (SK4) [26]. Among them, SK3 is the most abundant SK subunit expressed in ARH neurons [27, 28]. Importantly, selective deletion of SK3 from ARH Agouti-related peptide (AgRP) neurons increases firing frequency of AgRP neurons and sensitivity to diet-induced obesity in mice, and this is associated with chronic hyperphagia and decreased energy expenditure [29]. However, functions of SK3 in other ARH neurons on energy and glucose balance are not well studied.

In the present study, we re-analyzed the expression of SK genes and found abundant SK3 expression in POMC neurons. Then, we generated mice with SK3 specifically deleted in POMC neurons and analyzed metabolic phenotypes of these mice. We also compared SK current in POMC neurons and a subpopulation of POMC + ERα + neurons from male and female mice. Here we revealed a sexually dimorphic role of SK3 in POMC neuron functions on energy and glucose balance, which may involve the mechanism of a subpopulation of POMC + ERα + neurons.

## Materials and methods

### Secondary analysis of scRNA-Seq results

Count and Meta data were downloaded from GSE93374 and count data were normalized to counts per million (CPM). These data were filtered for cells in the ARH [30]. The count data were then joined to the meta data by cell ID, genes including SK1-4 and POMC were selected, and were exported to an Excel file. Further filtering was applied through Excel using metadata or expression data. We extracted the expression profiles of SK1-3 and POMC in neurons only. We calculated the number of SK neurons that express either only individual SK, both SK and POMC, both SK and AgRP, or all SK and POMC and AgRP, which resulted in four subpopulations of SK neurons: SK + POMC-AgRP-, SK + POMC + AgRP-, SK + POMC-AgRP + and SK + POMC + AgRP+. Meanwhile, we calculated the number of POMC neurons with or without the expression of each SK. We also compared the expression of SK3 in male and female POMC neurons.

### Mice

Care of all animals and procedures was approved by Baylor College of Medicine Institutional Animal Care and Use Committee. Mice were housed in a temperature-controlled room at 22–24 °C using a 12-h light, 12-h dark cycle. Regular chow (5V5R, PicoLab) and water were provided ad libitum.

Tamoxifen-inducible POMC-CreER^T2^ transgenic mice [31] were crossed with Rosa26-LSL-tdTomato (Jackson Laboratory, #007905) or with SK3^lox/lox^ mice [29, 32] (Jackson Laboratory, #019083) to generate Rosa26-LSL-tdTomato/POMC-CreER^T2^, SK3 ^lox/lox^/POMC-CreER^T2^ (referred as pomc-SK3 KO) and SK3^lox/lox^ (referred as controls) littermates. These mice received one dose of tamoxifen (Sigma, T5648) injection at 11 weeks of age (0.2 g/kg, i.p.), which induced Cre-recombinase activity specifically in mature POMC neurons. Further, ERα-ZsGreen mouse strain [33, 34] was crossed with Rosa26-LSL-tdTomato/POMC-CreER^T2^ mouse strain to generate POMC-CreER^T2^/Rosa26-LSL-tdTomato/ERα-ZsGreen mice for the electrophysiology recording of ERα-positive POMC neurons (POMC + ERα+) in the ARH.

### Validation of SK3^lox/lox^ recombination in POMC cells

Control and pomc-SK3 KO mice (after tamoxifen inductions) were anesthetized with inhaled isoflurane and euthanized. The pituitary, the ARH and the nucleus of the solitary tract (NTS) were collected. Genomic DNAs were extracted using the REDExtract-N-Amp Tissue PCR Kit (# XNATS; Sigma-Aldrich, St Louis, MO), followed by PCR amplification of the floxed or recombined SK3 alleles. Primer for floxed SK3: Forward 5’-AGG AGA GGG CTG ATT CTC AAG-3’ and Reverse 5’-GTA TCG GTG ACT GCT TCA TCC-3’; Primer for recombinant band of the floxed SK3: Forward 5’-CTT CCC ATA TAA CAG TGT CAG-3’ and Reverse 5’-GTA TCG GTG ACT GCT TCA TCC-3’.

### Serum corticosterone measurements

For the measurements of resting corticosterone, mice were rapidly anesthetized and decapitated at 9:00 am and the trunk blood was collected; to measure stress levels of corticosterone, mice were restrained for 60 min with a plastic restraint cone, and blood was collected from the tail vein. Plasma was obtained by centrifugation and assayed using a corticosterone ELIAS kit (EIACORT, Life Technologies Corporation, Grand Island, NY).

### Metabolic phenotypes

The body weight and the food intake were measured every 4 days. These mice were then put into TSE PhenoMaster metabolic cages at 5–6 months of age for 7 days to measure feeding and energy expenditure. Energy expenditure was analyzed using CalR analysis [35] with body weight as a covariate. Glucose tolerance test (GTT) and insulin tolerance test (ITT) were performed at 7 months of age. In GTT, overnight-fasted mice received intraperitoneal (i.p.) injections of D-glucose (1 mg/g body weight), and tail blood glucose was measured using a true-test glucometer immediately before and 15, 30, 60 and 120 min after injections. In ITT, 2 h-fasted mice received i.p. injections of 1 mU/g human insulin (Humulin R; Eli Lilly Corp., Indianapolis, IN), and blood glucose values were measured immediately before and 15, 30, 60 and 120 min after injections.

### Electrophysiological recordings

To label POMC neurons with tdTomato or ERα-ZsGreen for electrophysiological recordings, we generated double transgenic mouse POMC-CreERT2/Rosa26-LSL-tdTomato or triple transgenic mouse POMC-CreER^T2^/Rosa26-LSL-tdTomato/ERα-ZsGreen as described above. Four weeks after tamoxifen injection, mice were deeply anesthetized with isoflurane and transcardially perfused with a modified ice-cold sucrose-based cutting solution (pH 7.3) containing 10 mM NaCl, 25 mM NaHCO3, 195 mM Sucrose, 5 mM Glucose, 2.5 mM KCl, 1.25 mM NaH2PO4, 2 mM Na-Pyruvate, 0.5 mM CaCl2, and 7 mM MgCl2, bubbled continuously with 95% O2 and 5% CO2 [34]. The mice were then decapitated, and the entire brain was removed and immediately submerged in the cutting solution. Slices (250 μm) were cut with a Microm HM 650 V vibratome (Thermo Scientific). Three to four brain slices containing the ARH were obtained for each animal (bregma − 2.54 mm to − 1.46 mm; interaural 1.74 to 2.34 mm). The slices were recovered for 1 h at 34 °C and then maintained at room temperature in artificial cerebrospinal fluid (aCSF, pH 7.3) containing 126 mM NaCl, 2.5 mM KCl, 2.4 mM CaCl2, 1.2 mM NaH2PO4, 1.2 mM MgCl2, 5.0 mM glucose, and 21.4 mM NaHCO3) saturated with 95% O2 and 5% CO2 before recording. Slices were transferred to a recording chamber and allowed to equilibrate for at least 10 min before recording. The slices were superfused at 34 °C in oxygenated aCSF at a flow rate of 1.8-2 ml/min.

Whole-cell patch clamp recordings were performed in POMC neurons in the ARH visually identified by an upright microscope (Eclipse FN-1; Nikon) equipped with IR-DIC optics (×40 NIR;Nikon). Signals were processed using Multiclamp 700B amplifier (Axon Instruments), sampled using Digidata 1440 A, and analyzed offline on a PC with pCLAMP 10.3 (Axon Instruments). The brain slices containing ARH POMC neurons were bathed in oxygenated aCSF (32–34 °C) at a flow rate of approximately 2 ml/min [13, 36]. Patch pipettes with resistances of 3 to 5 MΩ were filled with solution containing: 126 mM K gluconate, 10 mM NaCl, 10 mM EGTA, 1 mM MgCl2, 2 mM Na-ATP, and 0.1 mMMg-GTP (adjusted to pH 7.3 with KOH) [13, 29]. Voltage clamp was used to record SK-like current in POMC neurons, as described before [13, 29, 37].

### RNAScope

Mice were anesthetized and perfused transcardially with 0.9% saline followed by 10% formalin. Brains were removed and post fixed in 10% formalin for 16 h at 4 °C and cryoprotected in 30% sucrose for 48 h. Brains were frozen, sectioned at 20 μm using the cryostat, and washed in DEPC-treated phosphate buffered saline (PBS, PH7.4) for 10 min. Sections were mounted on DEPC-treated charged slides, dried for 0.5 h at room temperature and stored at -80 °C. On the day of the RNAScope assay, the slides were thawed and then rinsed twice in PBS. Slides were baked in an oven for 30 min at 60 °C. After that, slides were post-fixed in 10% formalin for 15 min at 4 °C. Slides were then gradually dehydrated in ethanol (50%, 70% and 100%, 5 min each) and underwent target retrieval in RNAscope target retrieval reagents (322,001, ACDBio) for 5 min at 100 °C. Slides were incubated in protease III (#322,337, ACDBio) for 30 min at 40 °C. Slides were then rinsed in distilled water and incubated with RNAScope probes for *Pomc* (Mm-Pomc; #314,081-C3, ACDBio), *Esr1* (Mm-Esr1; #478,201-C2, ACDBio) and *Kcnn3* (Mm-Kcnn3; #427,961, ACDBio) for 2 h at 40 °C. Sections were then processed using the RNAScope Fluorescent Multiplex Detection Reagents (#320,851, ACDBio) according to the manufacturer’s instructions. Slides were cover-slipped and analyzed using a fluorescence microscope.

### Statistics

The minimal sample size was predetermined by the nature of the experiments and previous experience. For physiological readouts (body weight, food intake and GTT), 6–14 mice per group were included. For electrophysiological studies, 22–32 neurons from 3 different mice in each genotype or condition were included. For ELISA, 3–5 mice were included. The data are presented as mean ± SEM. Statistical analyses were performed using GraphPad Prism to evaluate normal distribution and variations within and among groups. Methods of statistical analyses were chosen based on the design of each experiment and are indicated in figure legends. P < 0.05 was considered to be statistically significant.

### Study approval

Care of all animals and procedures conformed to the Guide for Care and Use of Laboratory Animals of the US National Institutes of Health and were approved by the Institutional Animal Care and Use Committee of Baylor College of Medicine.

## Results

### SK3 is the most abundant SK gene in POMC neurons

**Fig. 1:**
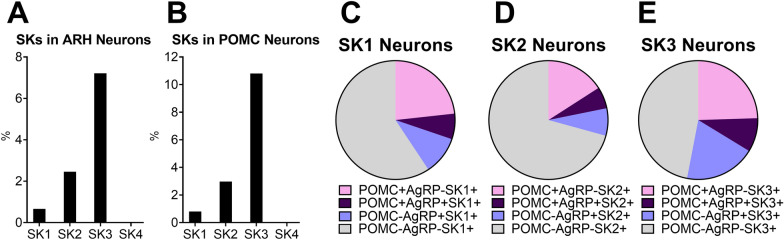
The expression of SKs in ARH neurons. **A** Percentage of neurons that express SK1, SK2, SK3 and SK4 in ARH. **B** Percentage of SK + POMC + neurons in POMC neurons. **C–E** Percentage of POMC + SK+, AgRP + SK + and POMC + AgRP + SK + neurons populations in SK neurons

We re-analyzed published single-cell RNA-Seq data of mouse hypothalamus [30] and mapped the four SK genes in hypothalamic POMC neurons. In ARH neurons, SK4 is scarcely expressed (Fig. 1 A), so we excluded SK4 from further analysis. Compared to SK1 and SK2, SK3 is the most abundant SK subunit in both ARH and POMC neurons (Fig. 1A–B). Close to 10% of POMC neurons co-express SK3 (Fig. 1B) and one third of SK3-expressing ARH neurons overlap with POMC neurons (Fig. 1E), both are higher than SK1 and SK2-expressing neurons (Fig. 1C–D). Interestingly, only 2/318 and 14/318 of SK3 + POMC + neurons co-express SK1 and SK2 respectively, suggesting a segregation among SK1, SK2 and SK3 in POMC neurons. Consistent with previous reports, SK3 is also highly expressed in AgRP neurons [27–29] (Fig. 1E). Interestingly, SK subunits are also co-expressed in a group of neurons that express both POMC and AgRP (Fig. 1C–E). SK3 has been demonstrated to mediate SK current in AgRP neurons and to inhibit AgRP neuron activity and functions [29]. Here, these results suggest that SK current in POMC neurons may be primarily mediated by SK3, and SK3 may also suppress POMC neurons functions.

### POMC-specific SK3 deficiency did not affect pituitary function

**Fig. 2:**
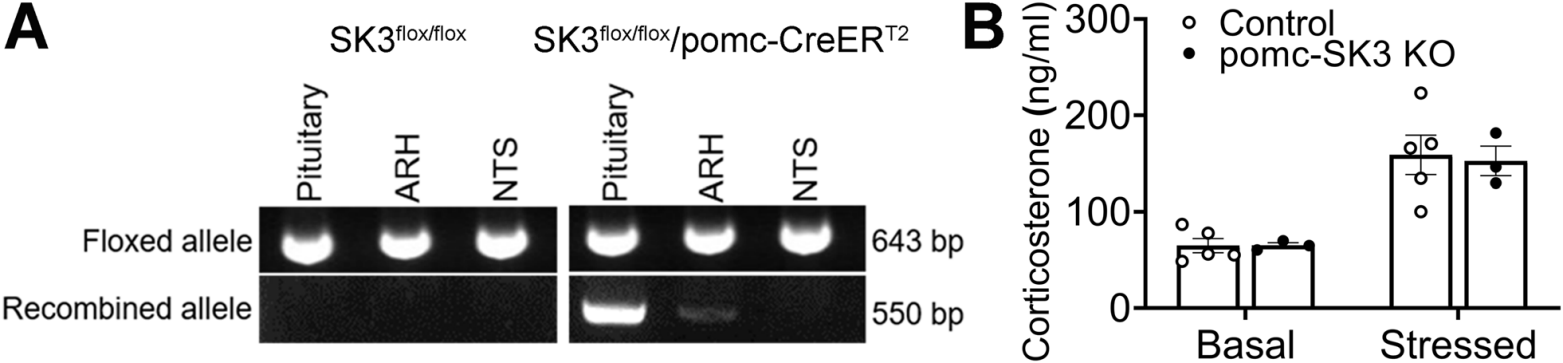
POMC specific deletion of SK3 didn’t affect pituitary function. **A** PCR detection of SK3^flox/flox^ allele and Cre-induced recombination in the ARH, NTS and pituitary from control or the pomc-SK3 KO mice. **B** Serum corticosterone level at basal and stressed conditions of male control and pomc-SK3 KO mice. Data are presented as mean ± SEM with individual data points. N = 3–5 mice per group

In order to examine the physiological functions of SK3 in POMC neurons, we crossed tamoxifen-inducible POMC-CreER^T2^ and SK3^lox/lox^ mice to generate pomc-SK3 KO (SK3^lox/lox^/POMC-CreER^T2^) and control (SK3^lox/lox^) mice. After tamoxifen induction, we validated the deletion of SK3 in POMC cells expressed in the ARH, NTS and pituitary. As expected, we detected a recombinant band of the floxed SK3 allele in the ARH, indicating the successful deletion of SK3 (Fig. 2A). Although POMC gene is also expressed in NTS neurons and regulates feeding [38, 39], we did not detect any recombinant band in the NTS, presumably due to fewer POMC neurons present in the NTS and/or the weak tamoxifen-induced Cre activity in these few NTS POMC neurons (Fig. 2A). Remarkably, we observed a strong recombinant band of the floxed SK3 allele in the pituitary (Fig. 2A), where POMC cells are abundant. Pituitary POMC cells predominately produce adrenocorticotropic hormone (ACTH), which induces adrenal corticosterone increase in response to stress [40–42]. To determine whether SK3 contributes to the function of pituitary POMC cells, we measured serum levels of corticosterone in the pomc-SK3 KO and control mice. POMC-specific SK3 deficiency did not change the serum corticosterone levels at either basal or stressed conditions (Fig. 2B). This result suggested that the deletion of SK3 in the pituitary POMC cells did not affect the hypothalamus-pituitary-adrenal axis (HPA). Thus, we used this pomc-SK3 KO mouse strain to investigate the function of SK3 in hypothalamic POMC neurons.

### POMC-specific SK3 deficiency did not change body weight in either male or female mice, but decreased feeding and physical activity in male mice

**Fig. 3:**
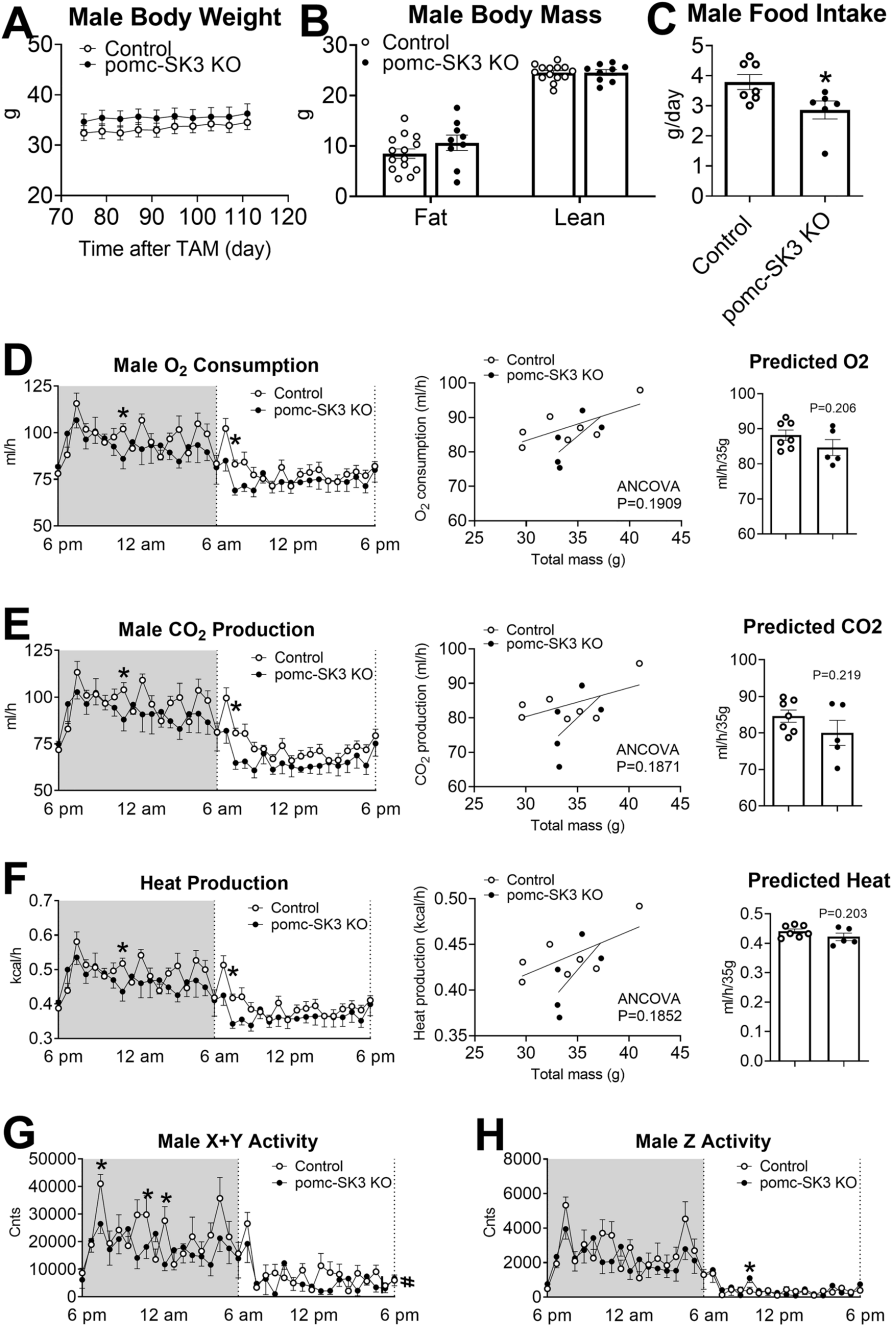
POMC specific deletion of SK3 decreased not only feeding but also physical activity in male mice, resulting in unchanged body weight. **A**-**C** Body weight (**A**), body mass (**B**) and food intake (**C**) of male control mice and pomc-SK3 KO mice. **D**-**H** O_2_ consumption (**D**), CO_2_ production (**E**) and heat production (**F**) of male control mice and pomc-SK3 KO mice. Left panel: temporal levels of each parameter measured by the TSE PhenoMaster during the dark cycle and light cycle within 24 h. Middle panel: ANCOVA analysis using body weight as a covariate (p value as indicated). Right panel: predicted O_2_ consumption, CO_2_ production or heat production using CalR analysis 35. Although two-way ANOVA did not reveal a significant difference, two-tailed unpaired t-tests detected several significantly different time points. **G**, **H** Physical activity (X + Y axis) (**G**) and physical activity (Z axis) (**H**) of male control mice and pomc-SK3 KO mice. Data are presented as mean ± SEM with individual data points. N = 6–14 mice per group. #P < 0.05 in two-way ANOVA analysis. *P < 0.05 in two-tailed unpaired t-test

We did not detect any difference in body weight between pomc-SK3 KO and control male mice (Fig. 3A). Consistently, neither fat mass nor lean mass was changed by POMC-specific SK3 deficiency (Fig. 3B). To fully characterize the metabolic phenotypes, we then put these mice into TSE PhenoMaster metabolic cages to measure feeding, energy expenditure and physical activity. Interestingly, in contrast to unchanged body weight, pomc-SK3 KO male mice showed significantly lower food intake than control male mice (Fig. 3C). Meanwhile, pomc-SK3 KO male mice showed a decrease trend of energy and heat production (Fig. 3D–F). pomc-SK3 KO male mice also showed significant lower X-/Y-axis activity (Fig. 3G) but unchanged Z-axis activity (Fig. 3H). Taken together, POMC-specific SK3 deficiency decreased not only energy intake but also physical activity in male mice, which ultimately reached a homeostatic balance to result in unchanged body weight.

**Fig. 4:**
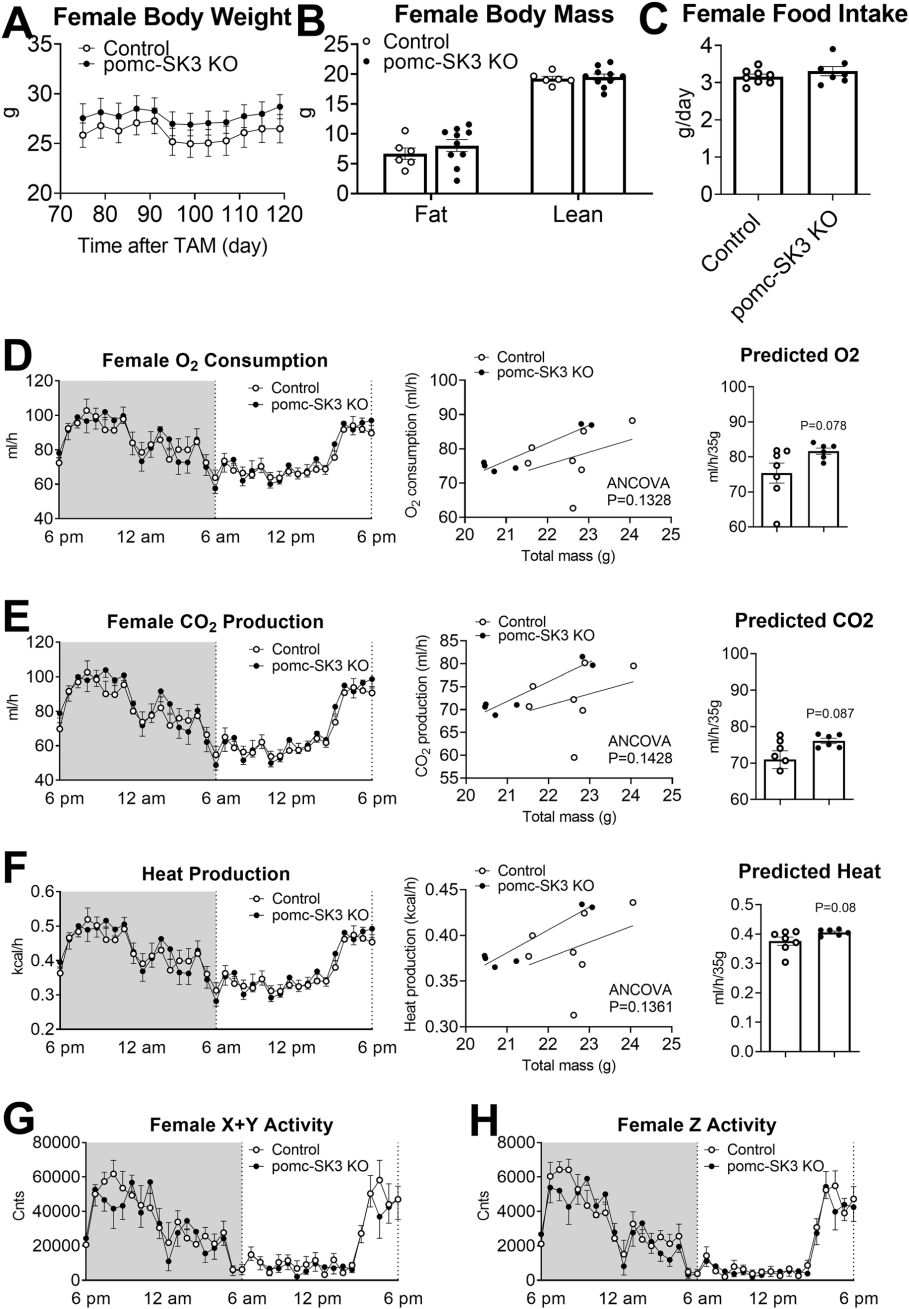
POMC specific deletion of SK3 did not regulate food intake, energy expenditure or body weight in female mice.** A** Body weight,** B** body mass,** C** food intake,** D** O_2_ consumption,** E** CO_2_ production,** F** heat production,** G** physical activity (X + Y axis) and** H** physical activity (Z axis) of female control mice and pomc-SK3 KO mice, the same reading as Fig. 3. Data are presented as mean ± SEM with individual data points. N = 6–14 mice per group

We also performed the same metabolic characterization in female mice, and found that female pomc-SK3 KO mice did not show any changes in body weight (Fig. 4A), fat or lean mass (Fig. 4B), food intake (Fig. 4C), energy expenditure (Fig. 4D–F) or physical activities (Fig. 4G, H). In summary, POMC-specific SK3 deficiency decreased feeding and physical activity only in male mice, without changing body weight balance. These results indicated that SK3 in POMC neurons plays a sexually dimorphic function in energy homeostasis without changing body weight.

### POMC-specific SK3 deficiency impaired glucose and insulin tolerance in female mice but not in male mice

**Fig. 5:**
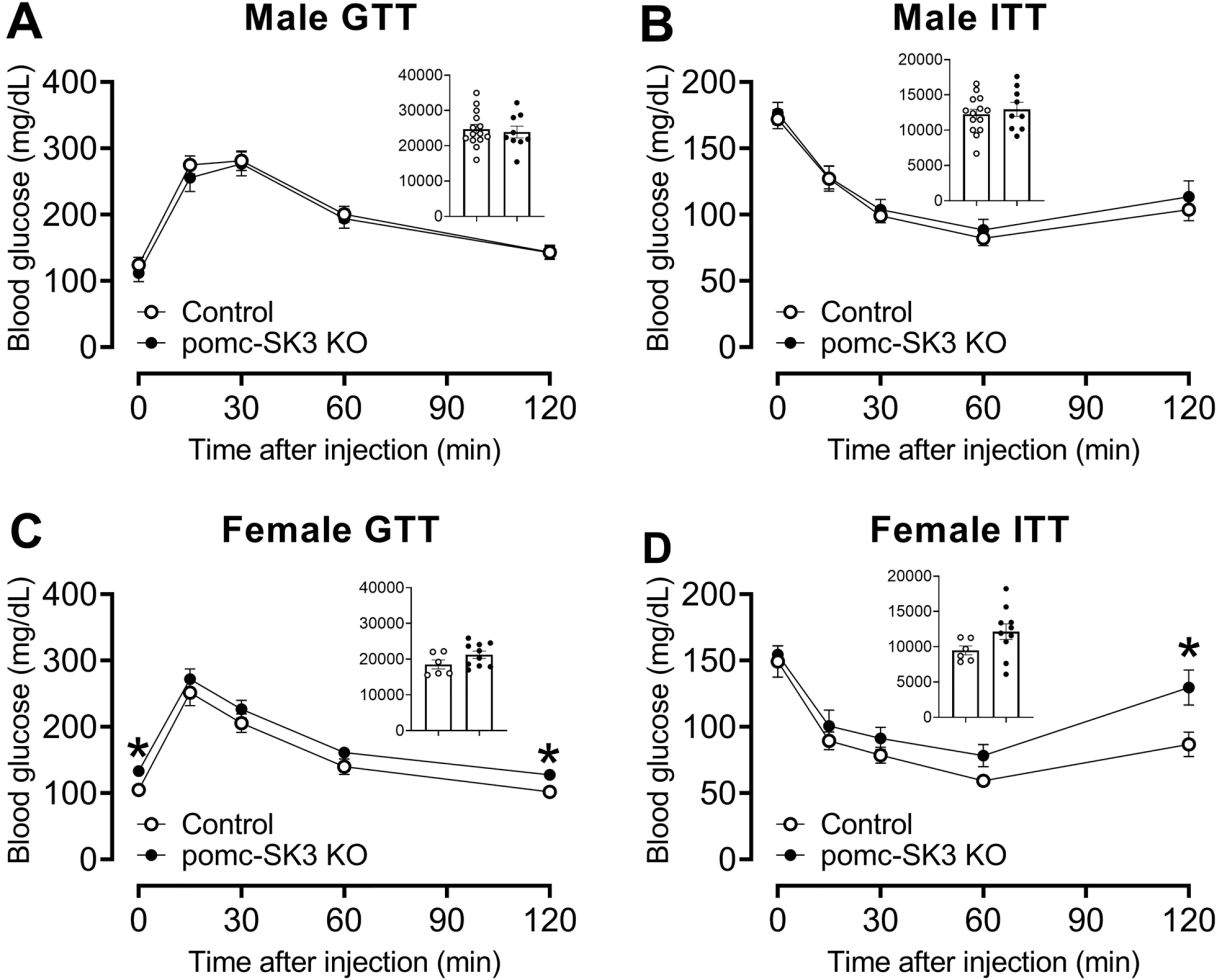
POMC specific deletion of SK3 impaired glucose and insulin tolerance in female mice but not in male mice. **A**, **C** GTT of male (**A**) and female (**C**) control and pomc-SK3 KO mice. **B**, **D** ITT of male (**B**) and female (**D**) control and pomc-SK3 KO mice. Inserted column figures are AUC analysis of GTT and ITT data, y axis is the arbitrary units for AUC. Data are presented as mean ± SEM with individual data points N = 6–14 mice per group. *, P < 0.05 in two-tailed unpaired t-test

We further tested whether POMC-specific SK3 deficiency also affects glucose balance in a sexually dimorphic manner. To this end, we performed GTT and ITT in age- and body weight- matched pomc-SK3 KO and control mice. Fed glucose, fasting glucose, GTT and ITT were comparable between male pomc-SK3 KO and control mice (Fig. 5A, B). These results indicated that SK3-expressing POMC neurons do not regulate glucose homeostasis in male mice. Despite unchanged body weight, pomc-SK3 KO female mice showed significant higher glucose at the beginning and the end of GTT test, indicating hyperglycemia and glucose intolerance (Fig. 5C). In ITT, pomc-SK3 KO female mice showed significant higher glucose 2 h after insulin injection, supporting impaired glucose balance (Fig. 5D). These results indicated that POMC-specific SK3 deficiency regulated glucose homeostasis but not energy homeostasis in female mice. Taken together, SK3 in POMC neurons plays a sexually dimorphic function in glucose homeostasis.

### Sex differences of SK channel in POMC neurons

**Fig. 6:**
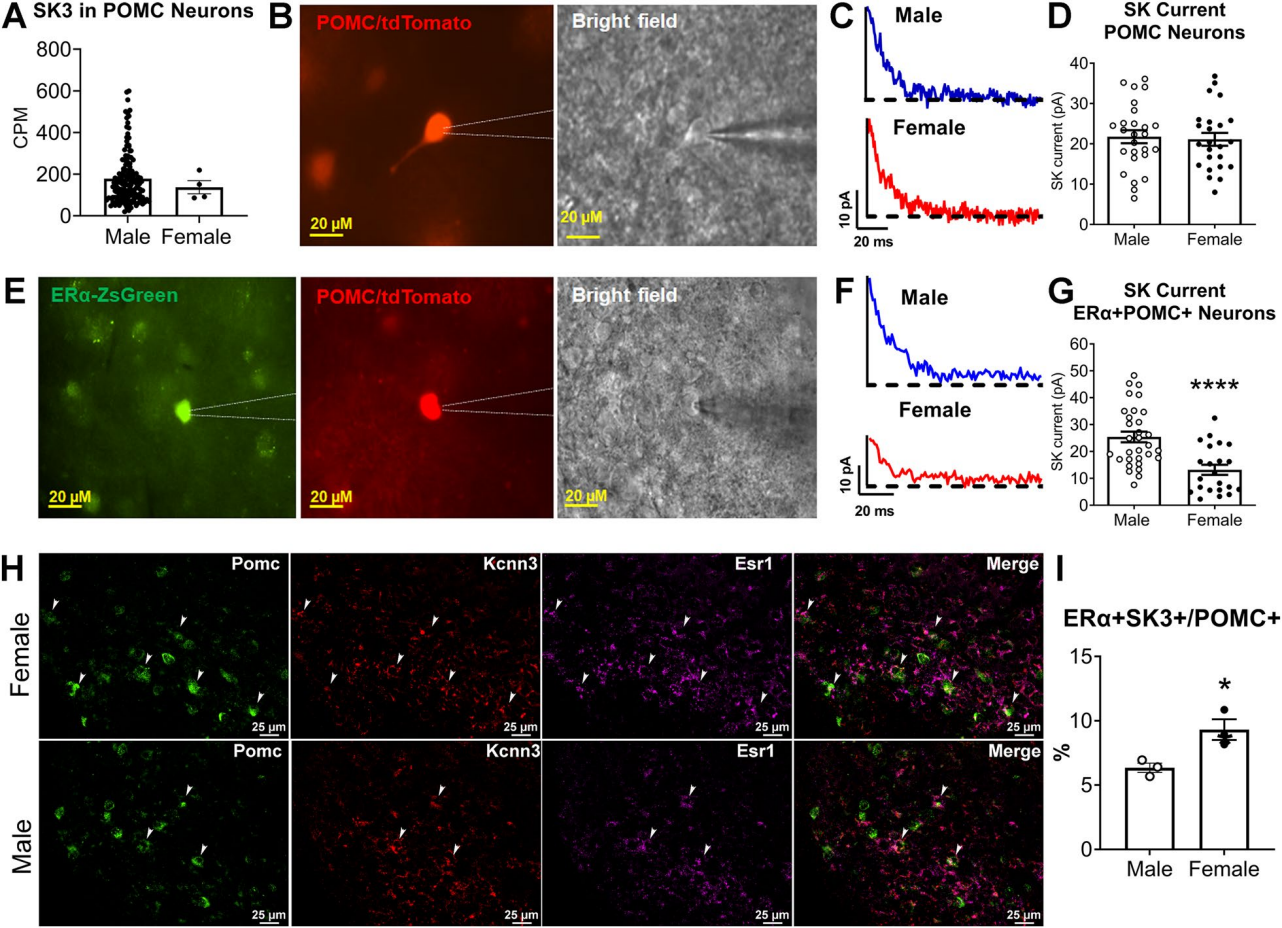
SK current and the expression of SKs in POMC neurons. **A** The expression of SK3 in male and female POMC neurons. **B** Fluorescence for tdTomato (left) and bright-field illumination (right) of a recorded POMC neuron in a brain slice. **C** Representative traces of SK current in male and female POMC neurons. **D** Quantification of SK current in POMC neurons from fed male and female mice. **E** Fluorescence for ZsGreen (left) and tdTomato (middle) and bright-field illumination (right) of a recorded POMC + ERα + neuron in a brain slice. **F** Representative traces of SK current in male and female POMC + ERα + neurons. **G** Quantification of SK current in POMC + ERα + neurons from male and female mice. **H** RNAScope using probes targeting POMC, SK3 and ERα in the ARH. White arrow indicated the neurons that co-express genes that encode POMC, SK3 and ERα. **I** Quantification of the percentage of POMC + SK3 + ERα + neurons out of total POMC neurons in the ARH. Data are presented as mean ± SEM with individual data points. N = 22–32 neurons per group. *, ****, P < 0.5 or 0.0001 in two-tailed unpaired t-test

We further explored the mechanisms underlying the sex differences of SK3 function in energy and glucose balance. We first re-analyzed published single-cell RNA-Seq data of mouse hypothalamus [30] to compare the expression of SK3 in male and female POMC neurons, and found there was no sex difference in SK3 mRNA levels (Fig. 6 A). Then we tested whether SK current is sexually dimorphic in POMC neurons. We labeled POMC neurons with tdTomato using POMC-CreER^T2^/Rosa26-LSL-tdTomato mice and used a SK current protocol [29, 37] (Fig. 6B) to record tdTomato-labeled POMC neurons (Fig. 6 C). We detected SK current in both male and female POMC neurons, but there was no sex difference (Fig. 6D). Previously we reported that POMC neurons are sexually dimorphic and more than half of POMC neurons are responsive to ERα agonist 13. Thus, we further recorded SK current in POMC + ERα + neurons (Fig. 6E) using POMC-CreER^T2^/Rosa26-LSL-tdTomato/ERα-ZsGreen triple transgenic mice 13. Surprisingly, male POMC + ERα + neurons showed significant higher SK current than female POMC + ERα + neurons (Fig. 6F, G). To further determine whether there is sexual dimorphism in the subpopulation of SK3 + POMC + ERα + neurons, we performed RNA scope with probes targeting SK3, ERα and POMC in the ARH (Fig. 6H). Our data showed that SK3 + POMC + ERα + triple positive neurons account for around 9% of total POMC neurons of ARH in female mice, which was higher than around 6% in male mice (Fig. 6I). These results suggests that ERα is a potential contributor to the sex differences observed in SK current and SK3 populations in POMC neurons. Male SK3 + POMC + ERα + neurons with higher SK current but lower population appear to contribute to energy balance, while female SK3 + POMC + ERα + neurons with lower current but higher population appear to regulate glucose balance.

## Discussion

We detected SK current in POMC neurons and found that SK3 is the dominant SK gene that may mediate SK current in POMC neurons. To explore the function of SK3 in POMC neuron functions, we used POMC-CreER^T2^ and SK3^lox^/^lox^ mice to generate mice with SK3 specifically deleted from mature POMC cells. Interestingly, although body weight was not changed, POMC-specific SK3 deficiency did decrease feeding and physical activity in male mice, which explained the unchanged body weight. Meanwhile, POMC-specific SK3 deficiency increased fasting glucose and impaired glucose and insulin tolerance only in female mice but not in male mice. Importantly, SK3 deletion is not detectable in NTS neurons, and POMC-specific SK3 deficiency did not affect the HPA axis, which suggested that these metabolic phenotypes could be attributed to hypothalamic POMC neurons. Further, SK current is higher but SK3-expressing neuron population is lower in male POMC + ERα + neurons. Together, we identified a sexually dimorphic role of SK3 in hypothalamic POMC neurons in both energy and glucose homeostasis, which was associated with the sex difference of SK current and the number of SK3-expressing neurons in a subpopulation of POMC + ERα + neurons.

Since SK channels trigger potassium outflux and SK3 deletion increases AgRP neuron activity and functions [29], it is expected that SK3 deletion also increases POMC neuron activity. In agreement with POMC neuron excitation, POMC-specific SK3 deficient male mice showed decreased food intake. However, POMC-specific SK3 deficient male mice also showed decreased physical activity and a decrease trend in heat production, which is inconsistent with previous documentation that POMC neurons promote energy expenditure and physical activity [1, 3]. This reduced physical activity counteracts reduced food intake, and as a result, body weight is not changed by POMC-specific SK3 deficiency in male mice. One possibility is that the reduced physical activity is a compensatory response to the reduced feeding behavior in order to conserve the energy storage. Another possibility is that it is a direct consequence of more active POMC neurons independent of feeding. Although it has never been reported that POMC neurons inhibit physical activity, recent reports suggested that POMC neurons are functionally heterogeneous even bidirectional in feeding. For example, POMC neurons not only release anorexic hormone α-MSH and ACTH, but also β-endorphin which plays a state-dependent role in feeding [43–46]. Acute central supplement of β-endorphin through intracerebroventricular injection stimulates feeding and antagonizes the effects of α-MSH on feeding, while chronic high dose of β-endorphin inhibits feeding [43]. On the other hand, only male mice but not female mice showed increased food intake and body weight gain with loss of β-endorphin without changing α-MSH level [44, 45]. In addition to POMC products, POMC neurons can also release neurotransmitters including glutamate and GABA [46]. Importantly, activation of cannabinoid receptor 1 (CB1R) in POMC neurons can promote feeding by increasing the release of β-endorphin [47] and GABA [48], which is opposite to the classic anorexic role of POMC neurons. So far, bidirectional functions of POMC neurons on energy expenditure and physical activity are not reported. Therefore, it is worth pursuing whether a subpopulation of POMC neurons, e.g., SK3-expressing POMC neurons, also inhibit energy expenditure and physical activity, which would represent a functionally segregated POMC population in the regulation of energy homeostasis.

Earlier studies showed that POMC neurons inhibit glycogenesis and improve insulin sensitivity [1, 4]. We showed that POMC-specific SK3 deficiency increased fasting glucose and caused glucose intolerance and insulin resistance in female mice. Consistent to this sexually dimorphic function of POMC neurons on glucose balance independent of body weight control, hypothalamic *Pomc* gene deficiency caused glucose and insulin intolerance only in non-obese juvenile female mice, which can be restored by re-expression of *Pomc* gene [49]. However, studies using the same mouse model showed that hypothalamic *Pomc* gene deficiency improves glucose tolerance despite massive obesity and insulin resistance in both obese and body weight-matched adult mice of both sexes [5]. This improvement of glucose tolerance is caused by reduced renal sympathetic nerve activity which leads to increased glycosuria, an insulin-independent mechanism [5, 6]. Hypothalamic POMC protein is processed to melanocortins including α-MSH and β-endorphin, which binds to melanocortin 4 receptor (MC4R) and mu-opioid receptors (MOR) respectively. Both MC4R-deficient mice and MOR-deficient mice show improved glucose tolerance despite obesity [50, 51]. MC4R-deficient mice develop insulin resistance without enhancement in beta cell function, and the improved glucose tolerance is attributed to glycosuria caused by reduced renal sympathetic nerve activity [50], similar to the mechanisms in POMC-deficient mice. Unlike MC4R-deficient mice, the improved glucose tolerance in MOR-deficient mice is contributed by increased glucose-stimulated insulin secretion (GSIS). Consistently, central activation of MOR causes decreases in GSIS and insulin sensitivity, and impairs glucose tolerance [52]. Together, POMC neurons can release α-MSH and β-endorphin to impair glucose tolerance through insulin-independent glycosuria and glucose-stimulated insulin-dependent mechanisms, respectively. Notably, it has been reported that disruption of GABA or glutamate release from POMC neurons does not change glucose tolerance in either male mice or female mice [53]. These results argue that POMC neurons and *Pomc* gene products differentially regulate glucose tolerance and insulin sensitivity through segregated mechanisms. These results suggested that the role of POMC neurons in glucose balance is more complicated than we expected. *Pomc* gene products regulate glucose differently in juvenile and adult, before and after the development of obesity, with different mechanisms between α-MSH and β-endorphin. Our observations that SK3 deficiency in POMC neurons causes glucose intolerance and insulin resistance in female mice are consistent with a possibility that enhanced POMC neuron activity leads to increased release of β-endorphin. However, it is not clear whether the POMC protein processing in SK3-expressing neurons is biased to the production of β-endorphin, and detailed mechanisms for the function of SK3 on POMC neuron functions and on glucose balance need to be further elucidated.

Accumulating evidence supports that POMC neurons are sexually dimorphic and functionally segregated not only in energy homeostasis [13–16], but also in glucose balance p^18^–^22^], which supports the functional heterogeneity of POMC neurons. We reported a subpopulation of SK3-expressing POMC neurons that regulate energy homeostasis in males and glucose balance in females, respectively. However, the detailed molecular mechanisms for this sexually dimorphic role of SK3-expressing POMC neurons are not clear. While the SK current and SK3 mRNA are comparable between total male and female POMC neurons, we found that female POMC neurons displayed significantly lower SK current in ERα + POMC + neurons but high population of SK + ERα + POMC neurons, which may contribute to the sexually dimorphic functions of SK3-expressing POMC neurons. Notably, we previously demonstrated that estrogen, by acting through ERα, can inhibit SK current in midbrain serotonin neurons [37], which explains the lower SK current in female SK + ERα + POMC neurons. Judging from the higher population of SK + ERα + POMC neurons in female mice, ERα may promote SK3 expression in POMC neurons. It is plausible that estrogen, acts on ERα, stringently regulating the function of SK3-expressing POMC neurons in female mice by inhibiting SK current and promoting SK3 expression, which contributes to the sexually dimorphic metabolic function of SK3 in POMC neurons. In addition, the functional SK channel also contains calmodulin (CaM), protein kinase CK2 and protein phosphatase 2 A (PP2A) [26], which may also contribute to the sex differences of SK current in POMC neurons. Unfortunately, current single-cell RNA-seq data resources especially from female mice are too restricted for the analysis of the sex differences and co-expression analysis of SK3 with ERα, CaM, CK2 and PP2A. One more plausible mechanism is that, compared to other POMC neuron population, SK + ERα + POMC neurons may project to or receive projections from distinct brain regions or neuron populations that contribute to the observed metabolic and sexually dimorphic phenotypes, which warrants future studies.

## Conclusion

We identified a sub-population of sexually dimorphic and functionally segregated POMC neurons that express SK3. SK3 in hypothalamic POMC neurons regulates energy homeostasis in male mice and glucose balance in female mice, respectively, without changing their body weight balance. Importantly, together with recent findings, the function of SK3 in POMC neurons challenges the conventional view that all POMC neurons universally prevent weight gain and reduce blood glucose, but support the heterogeneity of POMC neuron functions in energy and glucose balance.

## Data Availability

The data analysed in Fig. 1 are previously published and available from https://www.ncbi.nlm.nih.gov/geo/query/acc.cgi?acc=GSE93374.

## Abbreviations

POMC: Pro-opiomelanocortin
SK: Small conductance calcium-activated potassium channel
ERα: Estrogen receptor α
ARH: The arcuate nucleus of the hypothalamus
AHP: After-hyperpolarization
AgRP: Agouti-related peptide
CPM: Counts per million
HPA: The hypothalamus-pituitary-adrenal axis
CaM: Calmodulin
PP2A: Protein kinase CK2 and protein phosphatase 2 A
